# Development and validation of a nomogram for predicting chest drainage after CT-guided percutaneous lung biopsy: a multicenter retrospective study

**DOI:** 10.3389/fonc.2026.1930072

**Published:** 2026-10-12

**Authors:** Haiyu Liang, Yanyan Tang, Weicong Kang, Yanfeng Zhao

**Affiliations:** 1 Department of Diagnostic Imaging, Cancer Hospital, Chinese Academy of Medical Sciences, Peking, China; 2 Medical Imaging center, Liaocheng Tumor Hospital, Liaocheng, China; 3 Clinical Nutrition Department, Cancer Hospital, Chinese Academy of Medical Sciences, Peking, China

**Keywords:** chest drainage, clinical prediction model, CT-guided lung biopsy, external validation, nomogram, ridge regression

## Abstract

**Objective:**

To develop and externally validate a ridge logistic regression model containing only pre-procedural predictors to estimate the individual probability of requiring chest drainage after CT-guided percutaneous core-needle lung biopsy.

**Methods:**

This multicenter retrospective study included 2,492 patients. All 2,092 patients who underwent biopsy at Institution 1 from 2015 to 2025 were used for model development, and 400 patients treated at Institution 2 in 2025 formed the external-validation cohort. Nine variables available before needle entry were prespecified: age, sex, planned position, emphysema, lesion short-axis diameter, longitudinal lesion location, anatomical lobe, planned fissure traversal, and visceral-pleural adjacency. A ridge-penalized logistic model was fitted; the penalty was selected by stratified 10-fold cross-validation using the one-standard-error rule, and the complete modeling process was internally validated with 1,000 bootstrap resamples. The frozen model was applied directly to Institution 2 without refitting, recalibration, or threshold optimization. Development and external-validation sample sizes were formally assessed retrospectively using the Riley frameworks.

**Results:**

Chest drainage was required in 127/2,092 patients (6.1%) at Institution 1 and 34/400 (8.5%) at Institution 2. At Institution 1, the apparent AUC was 0.786 (95% CI, 0.745-0.827) and the optimism-corrected AUC was 0.766. For the frozen model at Institution 2, the AUC was 0.683 (95% CI, 0.579-0.779) and the Brier score was 0.0755 (95% CI, 0.0550-0.0982). The external calibration intercept was 0.41 (95% CI, 0.00-0.72), calibration slope was 1.13 (95% CI, 0.52-1.85), and observed-to-expected ratio was 1.44 (95% CI, 1.00-1.89), indicating systematic underprediction. The development sample was slightly smaller than required under conservative assumptions. Under the prespecified stringent precision targets, external validation would require approximately 17,397 patients and 1,479 events, whereas the available cohort contained 400 patients and 34 events. External validation should therefore be interpreted as independent but preliminary.

**Conclusion:**

The pre-procedural model showed moderate discrimination in an independent external institutional cohort but underestimated absolute risk. Prospective multicenter validation, with local recalibration where appropriate, is required before routine clinical use.

## 1 Introduction

CT-guided percutaneous core-needle biopsy (CT-PCNB) is widely used to obtain tissue from pulmonary lesions. Pneumothorax is a common complication, but only a subset of patients require chest drainage. Chest drainage entails an additional intervention, patient discomfort, prolonged observation or hospitalization, and potentially delayed subsequent treatment (1–4). A model targeting chest drainage may therefore be more useful for pre-procedural counseling and post-procedural observation planning than one predicting pneumothorax alone.

Previous studies have associated emphysema, lesion depth, fissure traversal, lesion size, and patient position with pneumothorax or chest drainage (2, 4–16). However, some previous models included procedural variables that were not strictly available at the intended prediction time, potentially introducing time-dependent bias or reverse causation and reducing the interpretability and implementability of pre-procedural prediction.

We therefore aimed to develop and externally validate a model restricted to information available before needle entry. All patients at Institution 1 were used for development; predictors were prespecified; ridge penalization and full-process bootstrap internal validation were used; and the frozen model was evaluated once at an independent institution. Performance was assessed by discrimination, calibration, overall prediction error, decision-curve analysis, and predicted-risk distributions.

## 2 Materials and methods

### 2.1 Study design and participants

This multicenter retrospective study was approved by the ethics committees of both participating centers (approval nos. 26/266-5990 and EC-20240112-1020), which waived the requirement for written informed consent for retrospective data use. Patients had provided procedural informed consent before biopsy, and data were anonymized before analysis.

Consecutive patients undergoing CT-PCNB at Institution 1 from January 2015 through December 2025 were screened. The external cohort comprised consecutive patients undergoing the procedure at Institution 2 in 2025.

Inclusion criteria were:

1. complete baseline clinical and imaging data;
2. post-procedural CT and chest-radiograph follow-up.

Exclusion criteria were:

1. incomplete clinical or imaging data;
2. absence of post-procedural imaging follow-up;
3. inability to cooperate with the required position or breath-hold instructions because of cognitive impairment or psychiatric illness.

The final sample comprised 2,092 patients from Institution 1 and 400 from Institution 2. All Institution 1 patients were used for model development; Institution 2 was used only for external validation of the frozen model. The patient was the unit of analysis, and each row represented one unique patient. Each patient was included once; for patients undergoing multiple lung biopsies, only the first biopsy meeting the eligibility criteria was included. Thus, there were no within-patient repeated observations and no adjustment for within-patient correlation was required. The participant flow is shown in **Figure 1**.

**Figure 1:**
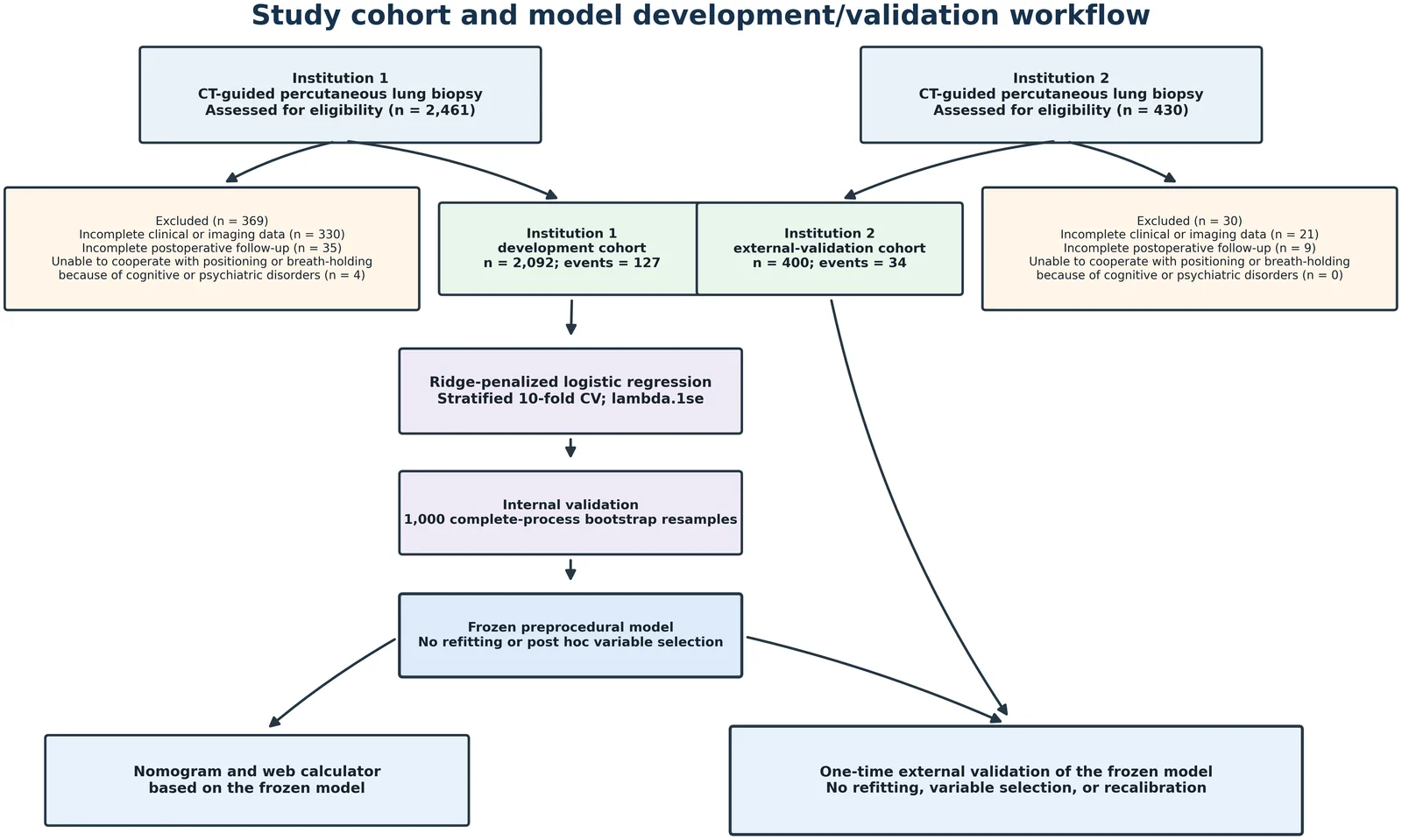
Patient flow. All eligible patients at Institution 1 were used for development and bootstrap internal validation; Institution 2 was used for external validation of the frozen model.

Among the 2,492 included patients, none of the nine prespecified predictors or chest-drainage outcomes were missing. Complete-case data were therefore used for development and validation, and multiple imputation was not required in the included cohorts. Exclusion before enrollment because of incomplete clinical data, missing or unevaluable pre-procedural CT, or absent post-procedural imaging may have introduced selection bias; where partial baseline data are available, characteristics of included and excluded patients should be compared.

### 2.2 Biopsy procedure and outcome

All biopsies were performed under sequential CT guidance with a 17-G coaxial introducer and an 18-G semiautomatic cutting needle; patient position and planned trajectory were determined by lesion location. Institution 1 used a Siemens SOMATOM Definition Edge CT scanner (64 rows/128 slices; 120 kV, 50 mA), and Institution 2 used a Neusoft NeuViz 128 Precision Plus scanner (64 rows/128 slices; 120 kV, 270 mA). At both centers, acquisition slice thickness was 3 mm and reconstructed slice thickness/increment was 1/1 mm. Institution 1 used a soft-tissue reconstruction algorithm and a window width/level of 350/15 HU; Institution 2 used a lung reconstruction algorithm and a window width/level of 400/40 HU. Apart from these scanner and reconstruction differences, the imaging measurement workflow was identical at both centers.

At both institutions, post-procedural monitoring comprised immediate limited chest CT to detect acute pneumothorax, manual compression of the puncture site for at least 30 min, and an upright expiratory chest radiograph 1-4 h after the procedure. Both centers used the same unchanged clinical rule for chest drainage throughout 2015-2025: all patients with >30% lung compression due to pneumothorax on radiography underwent mandatory closed chest drainage, whereas those with <=30% compression did not, even in the presence of severe symptoms, progressive pneumothorax, or respiratory impairment. All drainage procedures used small-bore indwelling catheters; no large-bore chest tubes were used. Needle aspiration without an indwelling catheter was not counted as an outcome event. Clinicians unaware of the candidate model predictors adjudicated the outcome according to these criteria.

### 2.3 Prediction time and predictors

The prediction time was defined as immediately before needle entry, after the operator had reviewed the pre-procedural CT and planned the trajectory; every predictor had to be available at that time.

Nine variables were prespecified: age (years), sex (female/male), planned patient position (supine/prone/lateral), visually assessed emphysema (absent/present), lesion short-axis diameter (mm), longitudinal lesion location (the lung divided equally into five craniocaudal regions: upper/upper-middle/middle/lower-middle/lower), anatomical lobe (LUL/LLL/RUL/RML/RLL), planned fissure traversal (no/yes), and visceral-pleural adjacency (lesion-to-visceral-pleura distance <=10 mm; no/yes). Short-axis diameter was the maximum short-axis dimension on the axial CT section showing the largest lesion area. Emphysema was visually defined as a focal low-attenuation area without a distinct wall and with reduced vascular markings on pre-procedural CT.

Imaging variables were measured retrospectively using prespecified operational definitions. To assess reproducibility of the binary visual emphysema assessment, 200 CT examinations were sampled in proportion to institutional cohort size (168 from Institution 1 and 32 from Institution 2). Two observers at each center completed two reading rounds one week apart. Case order was rerandomized before round 2; observers were blinded to their first-round readings, the other observer’s readings, and chest-drainage outcomes. Interobserver and intraobserver agreement were evaluated using unweighted Cohen’s kappa with patient-level bootstrap 95% CIs, together with raw, positive, and negative agreement. Detailed reproducibility results are provided in **Supplementary Table S1**.

This reproducibility study was limited to emphysema, the principal visually assessed imaging predictor. Because repeated readings were unavailable, formal interobserver or intraobserver analyses could not be performed for lesion diameter, longitudinal location, anatomical lobe, planned fissure traversal, or pleural adjacency; this limitation is addressed in the Discussion.

In round 1, pooled interobserver agreement was 96.5% (193/200), with Cohen’s kappa of 0.899 (bootstrap 95% CI, 0.818-0.968), positive agreement of 92.1%, and negative agreement of 97.7%. Round-2 interobserver agreement was 97.0% (kappa=0.913). Intraobserver agreement was 98.5% and 99.0% for the two observers (kappa=0.958 and 0.970, respectively). Round-1 interobserver kappa was 0.878 at Institution 1. The two observers at Institution 2 agreed on all 32 cases, but this result should be interpreted cautiously because of the small sample.

### 2.4 Statistical analysis and model development

Continuous variables are reported as median (interquartile range), and categorical variables as number (percentage). Standardized mean differences (SMDs) described case-mix differences between institutions. Cohorts were compared using the Mann-Whitney U test and Pearson chi-square test. All nine variables were retained regardless of univariable statistical significance. Age and lesion diameter were initially assessed as linear continuous terms and as natural cubic splines with 3 degrees of freedom; categorical variables were dummy-coded. Reporting followed the TRIPOD statement (17).

A ridge-penalized logistic regression model (alpha=0) was fitted using all Institution 1 patients (18). The penalty was selected by stratified 10-fold cross-validation and the one-standard-error rule (lambda.1se). The lambda.min model was used only as a sensitivity analysis and was not selected according to external performance. Internal validation used 1,000 bootstrap resamples. Within every resample, the design matrix was rebuilt, stratified 10-fold cross-validation was repeated to select lambda, and the ridge model was refitted. Optimism was estimated by comparing performance in each bootstrap sample with performance in the original Institution 1 data and subtracting mean optimism from apparent performance.

After development, all coefficients and the intercept were frozen. The model was applied directly to Institution 2 without coefficient refitting, recalibration, variable selection, or threshold optimization. Discrimination was assessed by AUC, overall prediction error by the Brier score, and calibration by the calibration intercept, calibration slope, observed-to-expected (O/E) ratio, and grouped calibration plots with bootstrap confidence intervals. Institution 1 was divided into 10 approximately equal predicted-risk groups (about 209–210 patients each). Given only 400 patients and 34 events at Institution 2, the external cohort was divided into five groups (80 patients each) to avoid excessive event sparsity. Patient-level bootstrap resampling (1,000 iterations) provided 95% CIs for observed group proportions and external performance metrics.

Clinical utility was assessed by decision-curve analysis over threshold probabilities of 1%-30%. A model-positive result was defined as triggering enhanced assessment of symptoms and vital signs after biopsy, closer imaging surveillance when needed, appropriately prolonged observation, and timely availability of chest-drainage equipment and personnel. The model was not intended to trigger prophylactic drainage or cancellation of biopsy. False positives could lead to additional monitoring or prolonged observation, whereas false negatives could delay recognition of patients who ultimately require drainage. The threshold range was descriptive and exploratory and does not represent recommended treatment thresholds.

Because this development and external-validation study used existing retrospective cohorts, sample size was determined by all eligible cases during the study period and no prospective sample-size calculation preceded data collection. Development sample size was formally assessed retrospectively using the Riley framework for binary-outcome prediction models (19). External-validation sample size was assessed using the Riley framework implemented in pmvalsampsize (20) for precise estimation of the calibration slope, O/E ratio, and C statistic. Analyses were performed in R 4.5.2, principally using glmnet and pROC. The formal retrospective sample-size assessments are detailed in **Supplementary Table S2**.

For clinical use, an interactive web calculator was developed in R Shiny (model version 1.0.0; https://liang0613.shinyapps.io/pre2/; accessed August 29, 2026). All nine predictors are required; no prediction is generated when any required input is missing, and the calculator performs no imputation. Five prespecified cases were used for online verification. The maximum absolute difference between downloaded web probabilities and probabilities calculated directly from the frozen R model was 2.78×10^–16^, below the prespecified tolerance of 10^-10^; displayed percentages differ only because of rounding. The interface is shown in **Supplementary Figure S1**. The five verification cases are provided in **Supplementary Table S3**.

## 3 Results

### 3.1 Patient and cohort characteristics

The study included 2,492 patients: 2,092 at Institution 1 and 400 at Institution 2. Chest drainage was required in 127 (6.1%) and 34 (8.5%) patients, respectively. None of the nine predictors or outcomes was missing among included patients.

Patient characteristics are presented in **Table 1**. Age, sex, emphysema, longitudinal location, anatomical lobe, fissure traversal, and pleural adjacency were generally similar. Lesions were substantially smaller at Institution 2 (median short-axis diameter, 10 mm [IQR, 7-18] vs 24 mm [IQR, 16-35]; SMD = 0.800). Planned-position distributions also differed, particularly the greater proportion of lateral positioning at Institution 2 (35.8% vs 12.2%; maximum level-specific SMD = 0.574). These case-mix differences should inform interpretation of external performance.

**Table 1:** Patient characteristics at the two institutions.

Variable	Level/statistic	Institution 1 (n=2,092)	Institution 2 (n=400)	SMD	P value
Age, years	Median (IQR)	62.0 (54.0–68.0)	62.0 (53.0–71.0)	0.058	0.224
Lesion short-axis diameter, mm	Median (IQR)	24.0 (16.0–35.0)	10.0 (7.0–18.0)	0.800	<0.001
Sex	Female	841 (40.2%)	175 (43.8%)	0.072	0.186
	Male	1251 (59.8%)	225 (56.2%)		
Planned patient position	Supine	620 (29.6%)	88 (22.0%)	0.574	<0.001
	Prone	1217 (58.2%)	169 (42.2%)		
	Lateral	255 (12.2%)	143 (35.8%)		
Emphysema	No	1629 (77.9%)	300 (75.0%)	0.068	0.209
	Yes	463 (22.1%)	100 (25.0%)		
Longitudinal lesion location	Upper	306 (14.6%)	75 (18.8%)	0.111	0.051
	Upper-middle	637 (30.4%)	103 (25.8%)		
	Middle	410 (19.6%)	70 (17.5%)		
	Lower-middle	410 (19.6%)	93 (23.2%)		
	Lower	329 (15.7%)	59 (14.8%)		
Anatomical lobe	LUL	585 (28.0%)	98 (24.5%)	0.132	0.074
	LLL	368 (17.6%)	67 (16.8%)		
	RUL	614 (29.3%)	124 (31.0%)		
	RML	96 (4.6%)	31 (7.8%)		
	RLL	429 (20.5%)	80 (20.0%)		
Planned fissure traversal	No	1899 (90.8%)	360 (90.0%)	0.026	0.626
	Yes	193 (9.2%)	40 (10.0%)		
Visceral-pleural adjacency	No	1332 (63.7%)	250 (62.5%)	0.024	0.656
	Yes	760 (36.3%)	150 (37.5%)		
Chest drainage	No	1965 (93.9%)	366 (91.5%)	0.094	0.070
	Yes	127 (6.1%)	34 (8.5%)		

Data are median (interquartile range) or number (percentage). For categorical variables, the maximum absolute level-specific SMD is reported in the first level row. P values are descriptive and were not used for predictor selection.

### 3.2 Model specification

Nonlinear modeling of age and lesion diameter did not significantly improve fit (likelihood-ratio chi-square=3.13, 4 df, P = 0.537); both were therefore retained as linear terms. The final ridge model comprised 16 parameters derived from nine prespecified variables. Complete frozen coefficients, coding, and reference categories are given in **Table 2**. Because these are penalized estimates, conventional Wald CIs and P values were not calculated, and the coefficients are not interpreted as independent or causal risk effects. The complete frozen specification is also reproduced in **Supplementary Table S4**.

**Table 2:** Frozen ridge-model coefficients, coding, and reference categories.

Predictor	Coding/comparison	Reference category/unit	Beta coefficient	exp(β)
Intercept	Baseline log odds	Female; supine; no emphysema; upper location; LUL; no fissure traversal; not pleura-adjacent	-4.3142	
Age	Per 1-year increase	Continuous, years	0.0175	1.0176
Lesion short-axis diameter	Per 1-mm increase	Continuous, mm	0.0014	1.0014
Sex	Male vs Female	Female	0.4289	1.5356
Planned patient position	Prone vs Supine	Supine	0.1333	1.1426
Planned patient position	Lateral vs Supine	Supine	0.1896	1.2088
Emphysema	Yes vs No	No	0.5979	1.8184
Longitudinal lesion location	Upper-middle vs Upper	Upper	0.1418	1.1523
Longitudinal lesion location	Middle vs Upper	Upper	-0.1853	0.8309
Longitudinal lesion location	Lower-middle vs Upper	Upper	0.1780	1.1948
Longitudinal lesion location	Lower vs Upper	Upper	0.2908	1.3376
Anatomical lobe	LLL vs LUL	LUL	-0.4372	0.6459
Anatomical lobe	RUL vs LUL	LUL	0.0170	1.0171
Anatomical lobe	RML vs LUL	LUL	-0.1264	0.8813
Anatomical lobe	RLL vs LUL	LUL	-0.0048	0.9952
Planned fissure traversal	Yes vs No	No	0.8956	2.4488
Visceral-pleural adjacency	Yes vs No	No	-0.9179	0.3994

Coefficients are from the final frozen ridge logistic model (lambda.1se=0.03257). exp(beta) is only a coefficient transformation and should not be interpreted as an independent risk-effect estimate; conventional Wald P values and confidence intervals are not reported.

Under the conservative assumptions, development required at least 2,533 patients and approximately 154 events; the available cohort of 2,092 patients and 127 events was slightly smaller. External validation required 4,139, 17,397, and 1,106 patients for precise estimation of the O/E ratio, calibration slope, and AUC, respectively. The governing criterion was the calibration slope: 17,397 patients and approximately 1,479 events. A sensitivity analysis using the observed external AUC of 0.683 increased the AUC-based requirement to 1,384 patients but did not change the overall requirement. The available external cohort of 400 patients and 34 events was substantially smaller than required for the prespecified stringent precision targets.

The linear predictor was LP=-4.31419 + 0.01748×age+0.001403×lesion short-axis diameter, plus the applicable dummy-variable coefficients in **Table 2**; the predicted probability was $p=\frac{1}{1+\exp\left(-LP\right)}$. A conventional point-based nomogram exactly transformed from the frozen model is shown in **Figure 2** (21, 22).

**Figure 2:**
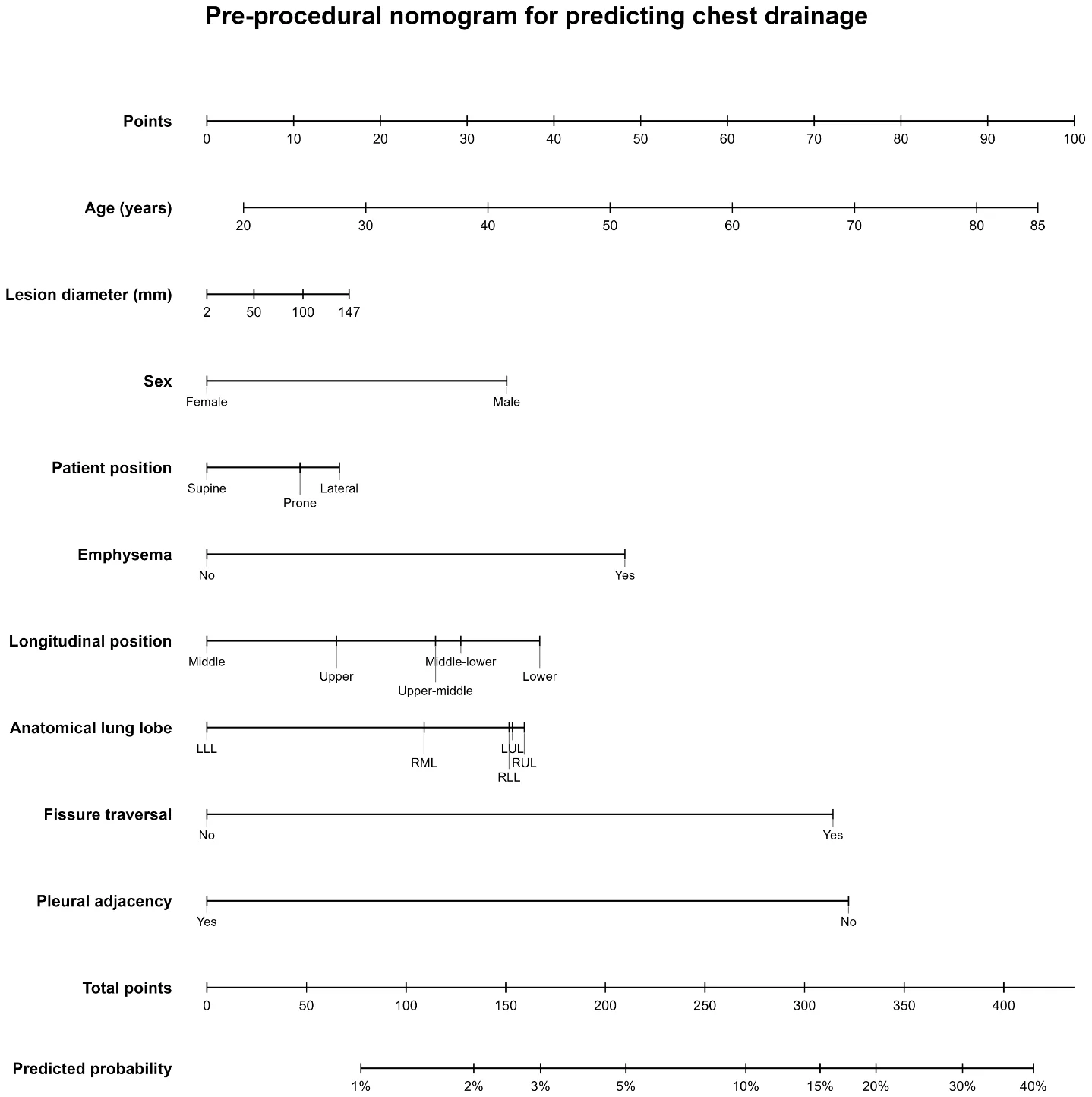
Pre-procedural nomogram for chest-drainage risk after CT-guided percutaneous core-needle lung biopsy. The nomogram is an exact point transformation of the final frozen fully linear ridge logistic model. Each predictor is assigned points, and total points map to predicted probability.

The penalty was selected by 10-fold cross-validation. The primary model used lambda.1se=0.03257, whereas lambda.min was used only for sensitivity analysis. The cross-validation curve and sensitivity comparison of the two lambda rules are presented in **Supplementary Figure S2** and **Supplementary Table S5**, respectively.

### 3.3 Model performance

At Institution 1, the apparent AUC was 0.786 (95% CI, 0.745-0.827) and the bootstrap optimism-corrected AUC was 0.766. The apparent and optimism-corrected Brier scores were 0.0525 and 0.0534, respectively. The apparent calibration intercept was approximately 0, the apparent calibration slope was 1.60, and the optimism-corrected calibration slope was 1.46.

When applied to Institution 2, the frozen model yielded an AUC of 0.683 (95% CI, 0.579-0.779) and a Brier score of 0.0755 (95% CI, 0.0550-0.0982). The calibration intercept was 0.41 (95% CI, 0.00-0.72), calibration slope 1.13 (95% CI, 0.52-1.85), and O/E ratio 1.44 (95% CI, 1.00-1.89). Mean predicted risk was 5.9%, below the observed event rate of 8.5%, indicating systematic underprediction of absolute risk. Confidence intervals were wide because only 34 external events occurred. ROC curves are shown in **Figure 3**, calibration plots in **Figure 4**, and performance metrics in **Table 3**. The corresponding external precision estimates are provided in **Supplementary Table S6**.

**Figure 3:**
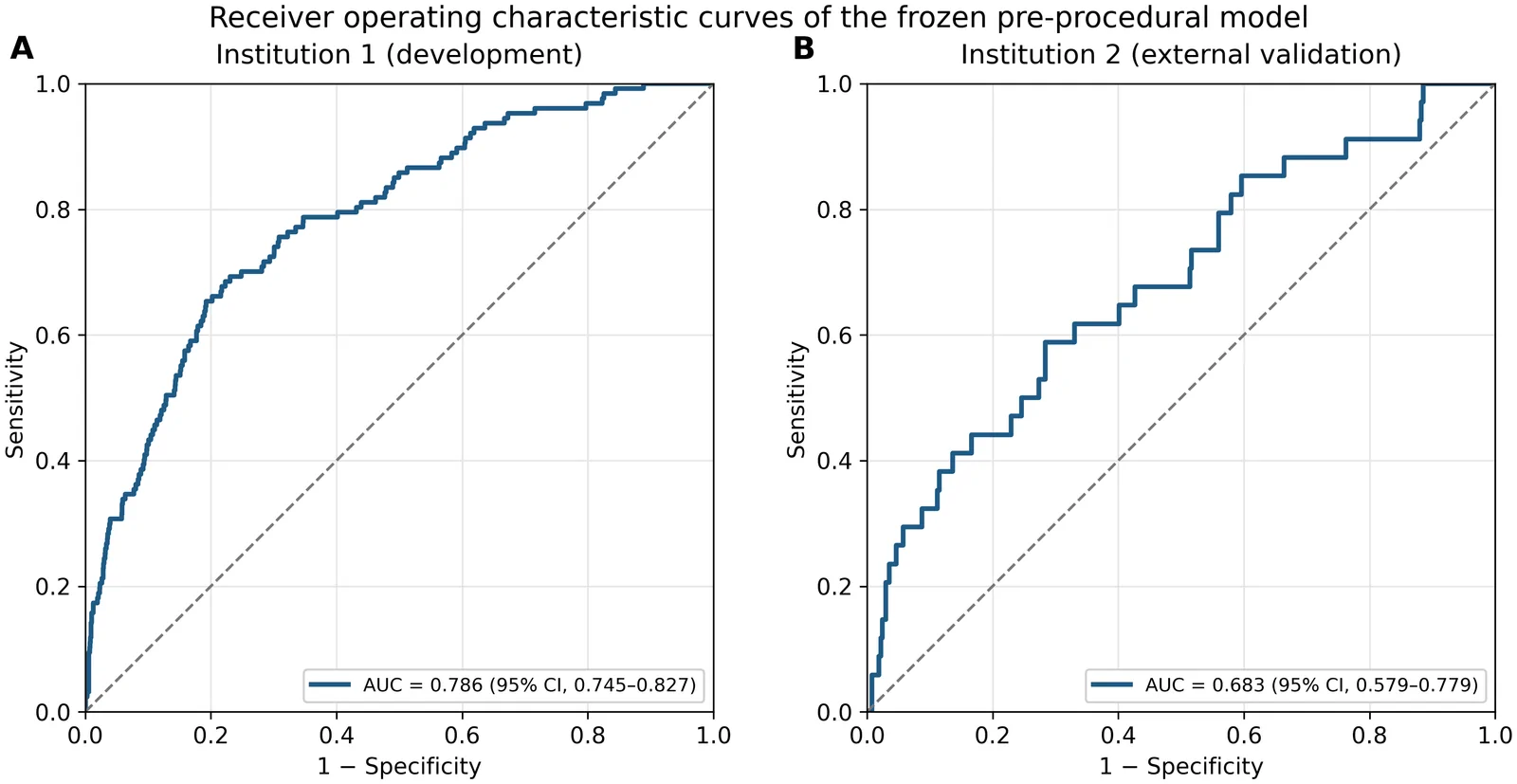
ROC curves for the frozen pre-procedural model: **(A)** Institution 1 development cohort and **(B)** Institution 2 external-validation cohort. The model was not refitted and no threshold was optimized at Institution 2.

**Figure 4:**
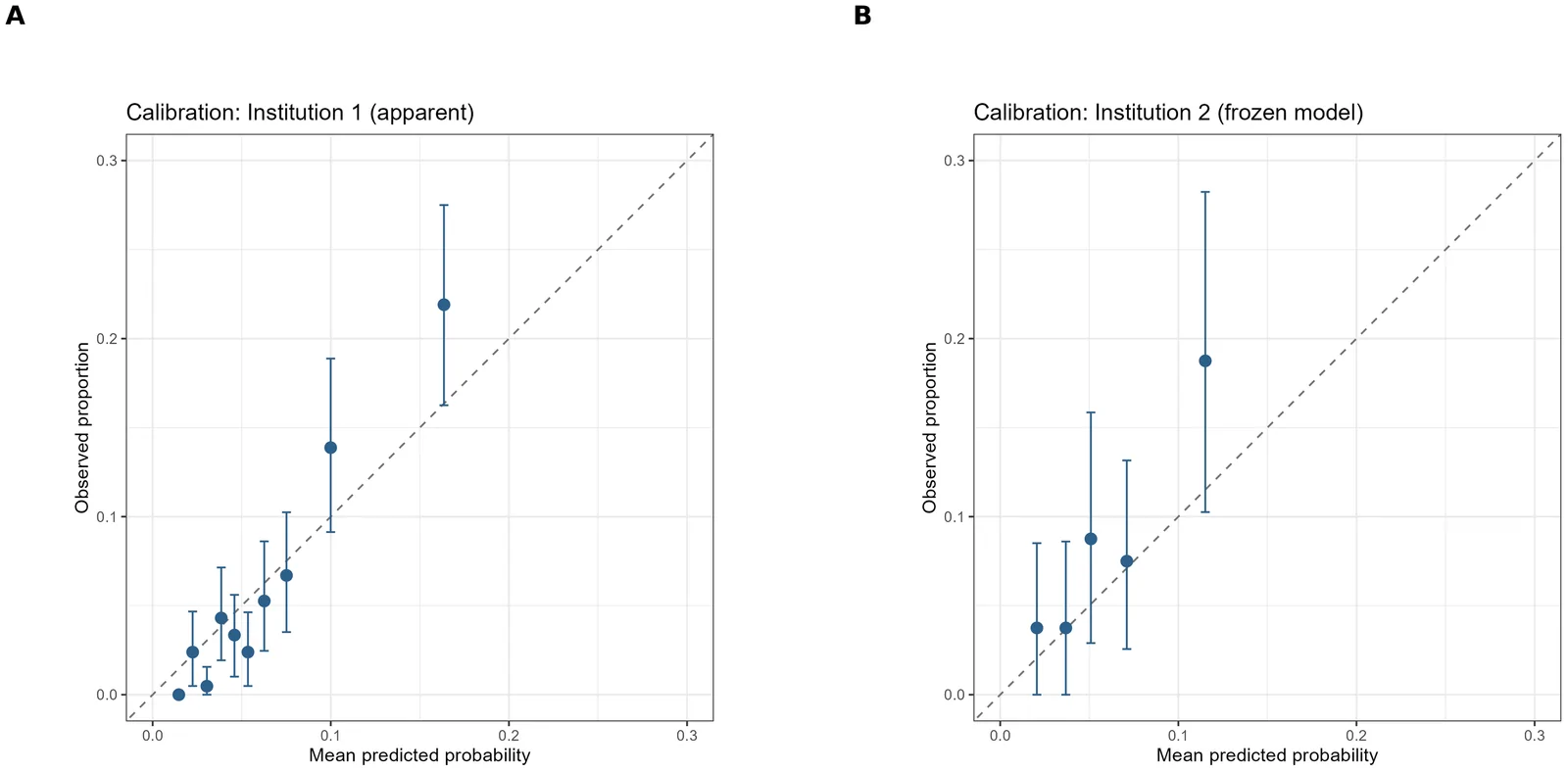
Calibration plots: **(A)** apparent grouped calibration at Institution 1 and **(B)** frozen-model calibration at Institution 2. Points show observed event proportions within predicted-risk quantiles, error bars show bootstrap 95% CIs, and the dashed line indicates perfect calibration.

**Table 3:** Model performance.

Metric	Institution 1 apparent	Institution 1 bootstrap-corrected	Institution 2 external validation (95% CI)
AUC	0.786 (0.745-0.827)	0.766	0.683 (0.579-0.779)
Brier score	0.0525	0.0534	0.0755 (0.0550-0.0982)
Calibration intercept	Approximately 0	Not estimated	0.41 (0.00-0.72)
Calibration slope	1.60	1.46	1.13 (0.52-1.85)
Observed/expected ratio	1.00	Not estimated	1.44 (1.00-1.89)
Mean predicted risk	6.1%	Not applicable	5.9% (5.5%-6.2%)
Observed event rate	6.1%	Not applicable	8.5%

Institution 2 metrics use the frozen model without refitting, recalibration, or threshold optimization. External 95% CIs were obtained by patient-level bootstrap resampling.

### 3.4 Clinical utility and predicted-risk distributions

Decision curves are shown in **Figure 5**. At Institution 1, the model had greater net benefit than either default strategy over most of the evaluated low-risk threshold range. At Institution 2, potential net benefit was mainly evident at thresholds of approximately 6%-17% and approached zero outside this range. Because these thresholds were not prespecified and no accepted clinical cutoff exists, the range is exploratory and is not a recommended decision threshold. Predicted-risk distributions overlapped between patients with and without chest drainage, especially at Institution 2, although risks were generally higher among patients who underwent drainage (**Supplementary Figure S3**).

**Figure 5:**
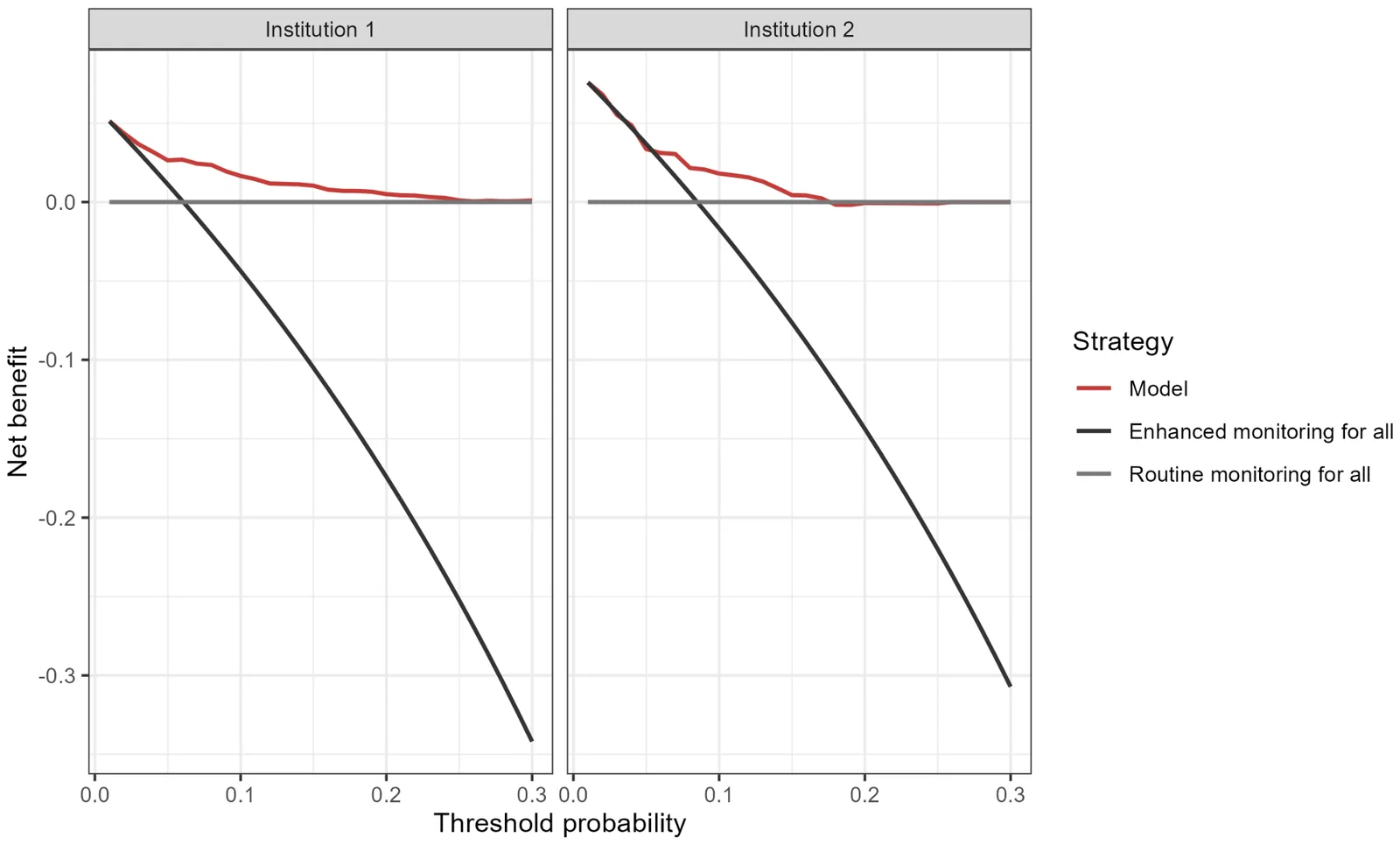
Decision-curve analysis comparing model-guided enhanced post-biopsy monitoring, enhanced monitoring for all patients, and routine monitoring for all patients. The external threshold range is exploratory and is not a recommended clinical cutoff.

All five online consistency tests passed. The maximum absolute difference between probabilities from the frozen R model and downloaded web probabilities was 2.78×10^-16^, below the prespecified tolerance of 10^-10^. Thus, the online implementation accurately reproduces the frozen equation; displayed percentages differ from exact probabilities only because of rounding. The interface accepts all nine pre-procedural predictors and displays the individual predicted probability and each variable’s contribution to the linear predictor.

## 4 Discussion

We developed a pre-procedural model for chest drainage after CT-PCNB and explicitly defined the prediction time as before needle entry. All Institution 1 patients were used for development, variables were prespecified rather than selected by significance, ridge shrinkage and full-process bootstrap internal validation were used, and the frozen model was evaluated once at Institution 2.

The model had useful but limited predictive ability. The optimism-corrected AUC was 0.766 at Institution 1 and declined to 0.683 externally, a clinically and methodologically important decrease. The external cohort differed in lesion size and planned position. Patient-level procedure dates or calendar years were not retained in the analytical database, precluding assessment of temporal trends or performance drift at Institution 1 during 2015-2025. Although the clinical drainage rule was unchanged at both centers, unmeasured changes in equipment, operator experience, patient selection, or periprocedural care cannot be excluded.

External calibration showed systematic underprediction: mean predicted risk was 5.9% versus an observed event rate of 8.5%; the positive calibration intercept and O/E ratio of 1.44 supported this finding. Although the calibration-slope estimate was near 1, only 34 external events occurred and the CI was wide. External validation should therefore be regarded as preliminary. Updating the intercept through local recalibration might improve absolute-risk estimates, but recalibration was deliberately not performed so that the original frozen model could be evaluated faithfully.

In the final ridge model, older age, larger lesion diameter, male sex, emphysema, and planned fissure traversal contributed to higher predicted probability, with relatively large contributions from fissure traversal and emphysema. Age may summarize lung elasticity, tissue fragility, re-expansion capacity, and unmeasured comorbidity or physiological reserve. Older age therefore increased predicted risk but may be a proxy for correlated physiological features rather than a direct cause (11, 12). Larger lesion diameter also slightly increased predicted risk, but its coefficient was near zero, indicating limited incremental information after accounting for the other predictors (3, 5, 8, 12, 14).

The association of emphysema with higher predicted risk is clinically plausible. Damaged alveolar architecture and reduced elastic recoil may impede spontaneous closure of an alveolar-pleural fistula, increasing persistent air leakage or progressive pneumothorax. Even with similar initial pneumothorax, patients with emphysema may more readily progress to requiring drainage (8, 10, 12–14). Interobserver and intraobserver agreement in 200 patients was high, supporting reproducible model application. Reproducibility, however, does not establish a causal relationship.

Planned fissure traversal was among the variables with the largest relative predictive contributions. A planned trajectory across a fissure may involve an additional visceral-pleural interface, increasing pleural and parenchymal injury and persistent air leakage (4, 8, 12–14). The model uses the trajectory planned on pre-procedural CT, not the actual trajectory confirmed after needle entry. This variable is therefore relevant to pre-procedural stratification and planning, but does not imply that every planned fissure traversal will require drainage. Operators may consider an alternative trajectory when compatible with safe, diagnostically successful sampling, but the model cannot replace procedural judgment.

Male sex contributed to higher predicted risk, although the mechanism is uncertain. Sex may capture body habitus, lung volume, smoking exposure, parenchymal change, or other incompletely measured features; it is predictive information rather than a modifiable independent risk factor (8, 12, 14). Pleural adjacency contributed to lower predicted risk, possibly because such lesions require traversal of less aerated lung, reducing parenchymal and alveolar injury. Nonadjacent lesions may require a longer intrapulmonary path and provide more opportunity for air leakage (5, 8, 9, 12–14). Pleural adjacency is not equivalent to actual needle-path length, and risk may also depend on entry angle, number of pleural punctures, respiratory motion, and technique.

Patient position, longitudinal location, and anatomical lobe made varying contributions, without a simple linear or monotonic pattern across categories. These variables may jointly capture thoracic anatomy, lobar mobility, lesion accessibility, and planned-trajectory differences (8, 12, 14–16). Several lobe coefficients were near zero, indicating little additional information relative to the reference category. Procedural strategy should therefore not be based on any single coefficient; all predictors should be entered into the frozen model to obtain an integrated individual estimate.

The primary aim was individualized prediction, not identification of independent risk factors through hypothesis testing (17). Ridge regression reduces overfitting by shrinking all coefficients toward zero (18); exponentiated coefficients indicate the direction and relative size of predictive contributions, not odds ratios from an unpenalized multivariable model, and cannot establish statistical significance. Predictive association also does not demonstrate causation. The model is best suited to integrated pre-procedural assessment, patient communication, and allocation of post-biopsy monitoring resources, not to decisions based on one variable to cancel biopsy, alter treatment, or place a prophylactic drain.

Decision-curve analysis suggested potential net benefit from model-guided enhanced monitoring over a limited low-threshold range, mainly 6%-17% at Institution 2. For higher predicted risks, appropriate actions are enhanced observation, longer monitoring, and greater preparedness to manage pneumothorax rather than prophylactic drainage. Because the relative costs of unnecessary prolonged monitoring and delayed recognition have not been formally quantified, these thresholds are exploratory and require prospective clinical evaluation.

This study has limitations. First, the retrospective design and complete-record eligibility requirement may have introduced selection bias; patients excluded for incomplete clinical or imaging data cannot be reliably recovered by multiple imputation. Second, 2,092 development patients and 127 events were slightly below the conservative requirement; shrinkage, prespecification, and full-process bootstrapping reduce and quantify overfitting but cannot fully compensate for limited events. Third, the external cohort had only 400 patients and 34 events, versus approximately 17,397 patients and 1,479 events under stringent precision targets; calibration-slope and O/E intervals were wide, making this an independent but preliminary validation. Fourth, patient-level dates were unavailable, precluding assessment of temporal heterogeneity and performance drift. Fifth, despite high emphysema reproducibility, the one-week interval may not completely exclude recall, and reproducibility of other imaging predictors and emphysema severity was not evaluated. Sixth, smoking history, tract-sealing techniques, and other potential factors were unavailable or incompletely standardized. Finally, validation was limited to one external institution; larger, geographically diverse prospective validation and impact studies are needed before routine use.

## 5 Conclusions

A pre-procedural ridge model based on nine routinely available variables showed moderate discrimination for the individual probability of chest drainage after CT-PCNB. External validation demonstrated systematic underprediction and substantial uncertainty. The model and nomogram may support research and structured pre-procedural risk communication, but prospective multicenter validation and, where appropriate, local recalibration are required before routine deployment.

## Data Availability

The original contributions presented in the study are included in the article/**Supplementary Material**, further inquiries can be directed to the corresponding author/s.

## References

[1] Haaga JR Alfidi RJ . Precise biopsy localization by computer tomography. Radiology. (1976) 118:603–7. doi: 10.1148/118.3.603 1251009

[2] Heerink WJ de Bock GH de Jonge GJ Groen HJ Vliegenthart R Oudkerk M . Complication rates of CT-guided transthoracic lung biopsy: meta-analysis. Eur Radiol. (2017) 27:138–48. doi: 10.1007/s00330-016-4357-8 27108299 PMC5127875

[3] Yang L Liang T Du Y Guo C Shang J Pokharel S . Nomogram model to predict pneumothorax after computed tomography-guided coaxial core needle lung biopsy. Eur J Radiol. (2021) 140:109749. doi: 10.1016/j.ejrad.2021.109749 34000599

[4] Hiraki T Mimura H Gobara H Shibamoto K Inoue D Matsui Y . Incidence of and risk factors for pneumothorax and chest tube placement after CT fluoroscopy-guided percutaneous lung biopsy: retrospective analysis of the procedures conducted over a 9-year period. AJR Am J Roentgenol. (2010) 194:809–14. doi: 10.2214/AJR.09.3224 20173164

[5] Yeow KM Su IH Pan KT Tsay PK Lui KW Cheung YC . Risk factors of pneumothorax and bleeding: multivariate analysis of 660 CT-guided coaxial cutting needle lung biopsies. Chest. (2004) 126:748–54. doi: 10.1378/chest.126.3.748 15364752

[6] Zhao Y Wang X Wang Y Zhu Z . Logistic regression analysis and a risk prediction model of pneumothorax after CT-guided needle biopsy. J Thorac Dis. (2017) 9:4750–7. doi: 10.21037/jtd.2017.09.47 29268546 PMC5721042

[7] He C Zhao L Yu HL Zhao W Li D Li GD . Pneumothorax after percutaneous CT-guided lung nodule biopsy: a prospective, multicenter study. Quant Imaging Med Surg. (2024) 14:208–18. doi: 10.21037/qims-23-891 38223129 PMC10784109

[8] Huo YR Chan MV Habib AR Lui I Ridley L . Pneumothorax rates in CT-guided lung biopsies: a systematic review and meta-analysis of risk factors. Br J Radiol. (2020) 93:20190866. doi: 10.1259/bjr.20190866 31860329 PMC7362905

[9] Lee HY Lee IJ . Assessment of independent risk factors of developing pneumothorax during percutaneous core needle lung biopsy: focus on lesion depth. Iran J Radiol. (2016) 13(4):e30929. doi: 10.5812/iranjradiol.30929 27895865 PMC5116566

[10] Zhou M Wang T Wei D Zhu Y Jiang Y Zuo C . Incidence, severity and tolerability of pneumothorax following low-dose CT-guided lung biopsy in different severities of COPD. Clin Respir J. (2021) 15:84–90. doi: 10.1111/crj.13272 32935471

[11] Bie ZX Guo RR Li B Wang CE Xu S Li YM . Analysis of pneumothorax occurrence and influencing factors in elderly patients undergoing CT-guided percutaneous lung biopsy. J Zhonghua Lao Nian Yi Xue Za Zhi. (2024) 43:1575–80. doi: 10.3760/cma.j.issn.0254-9026.2024.12.01130704229

[12] Ruud EA Stavem K Geitung JT Borthne A Søyseth V Ashraf H . Predictors of pneumothorax and chest drainage after percutaneous CT-guided lung biopsy: a prospective study. J Eur Radiol. (2021) 31:4243–52. doi: 10.1007/s00330-020-07449-6 33354745

[13] Kakizawa H Toyota N Hieda M Hirai N Tachikake T Matsuura N . Risk factors for severity of pneumothorax after CT-guided percutaneous lung biopsy using the single-needle method. J Hiroshima J Med Sci. (2010) 59:43–50. 21265263

[14] Zhao Y Bao D Wu W Tang W Xing G Zhao X . Development and validation of a prediction model of pneumothorax after CT-guided coaxial core needle lung biopsy. J Quant Imaging Med Surg. (2022) 12:5404–19. doi: 10.21037/qims-22-176 36465829 PMC9703113

[15] O'Neill AC McCarthy C Ridge CA Mitchell P Hanrahan E Butler M . Rapid needle-out patient-rollover time after percutaneous CT-guided transthoracic biopsy of lung nodules: effect on pneumothorax rate. J Radiol. (2012) 262:314–9. doi: 10.1148/radiol.11103506 22106349

[16] Kinoshita F Kato T Sugiura K Nishimura M Kinoshita T Hashimoto M . CT-guided transthoracic needle biopsy using a puncture site-down positioning technique. AJR Am J Roentgenol. (2006) 187:926–32. doi: 10.2214/AJR.05.0226 16985136

[17] Collins GS Reitsma JB Altman DG Moons KGM . Transparent reporting of a multivariable prediction model for individual prognosis or diagnosis (TRIPOD): the TRIPOD statement. Eur J Clin Invest. (2015) 45:204–14. doi: 10.1111/eci.12376 25623047

[18] Friedman J Hastie T Tibshirani R . Regularization paths for generalized linear models via coordinate descent. J Stat Softw. (2010) 33:1–22. doi: 10.18637/jss.v033.i01 PMC2929880 20808728

[19] Riley RD Snell KI Ensor J Burke DL Harrell FE Jr Moons KG . Minimum sample size for developing a multivariable prediction model: PART II - binary and time-to-event outcomes. Stat Med. (2019) 38:1276–96. doi: 10.1002/sim.7992 30357870 PMC6519266

[20] Riley RD Debray TPA Collins GS Archer L Ensor J van Smeden M . Minimum sample size for external validation of a clinical prediction model with a binary outcome. Stat Med. (2021) 40:4230–51. doi: 10.1002/sim.9025 34031906

[21] Balachandran VP Gonen M Smith JJ DeMatteo RP . Nomograms in oncology: more than meets the eye. Lancet Oncol. (2015) 16:e173–80. doi: 10.1016/S1470-2045(14)71116-7 25846097 PMC4465353

[22] Iasonos A Schrag D Raj GV Panageas KS . How to build and interpret a nomogram for cancer prognosis. J Clin Oncol. (2008) 26:1364–70. doi: 10.1200/JCO.2007.12.9791 18323559

