# Supplementary material for: Development and validation of a nomogram for predicting chest drainage after CT-guided percutaneous lung biopsy: a multicenter retrospective study
Source: Front Oncol. 2026 Oct 12;16:1930072. doi: 10.3389/fonc.2026.1930072 (PMC13696289; doi:10.3389/fonc.2026.1930072)
Supplement: Supplementary file 1 [file Table1.docx]

## Supplementary Table S1. Reproducibility of visual emphysema assessment

| **Comparison** | **n** | **Overall agreement** | **Cohen’s κ** | **Additional agreement** |
| --- | --- | --- | --- | --- |
| Pooled interobserver, round 1 | 200 | 96.5% (193/200) | 0.899 (bootstrap 95% CI, 0.818–0.968) | Positive 92.1%; negative 97.7% |
| Pooled interobserver, round 2 | 200 | 97.0% | 0.913 | — |
| Intraobserver, observer A | 200 | 98.5% | 0.958 | — |
| Intraobserver, observer B | 200 | 99.0% | 0.970 | — |
| Institution 1 interobserver, round 1 | 168 | — | 0.878 | — |
| Institution 2 interobserver, round 1 | 32 | 100% | Not estimable/degenerate agreement | Interpret cautiously because n=32 |

Two observers at each institution completed two blinded rounds one week apart. Case order was rerandomized before round 2. This formal reproducibility study was limited to emphysema; repeated readings for the other imaging predictors were unavailable.
