# Supplementary material for: Development and validation of a nomogram for predicting chest drainage after CT-guided percutaneous lung biopsy: a multicenter retrospective study
Source: Front Oncol. 2026 Oct 12;16:1930072. doi: 10.3389/fonc.2026.1930072 (PMC13696289; doi:10.3389/fonc.2026.1930072)
Supplement: Supplementary file 2 [file Table2.docx]

## Supplementary Table S2. Formal sample-size assessment

| **Component** | **Criterion** | **Required sample** | **Available sample** |
| --- | --- | --- | --- |
| Development | Conservative Riley assessment | 2,533 patients; ~154 events | 2,092 patients; 127 events |
| External validation | O/E precision | 4,139 patients | 400 patients; 34 events |
| External validation | Calibration-slope precision | 17,397 patients; ~1,479 events | 400 patients; 34 events |
| External validation | AUC precision, anticipated AUC 0.766 | 1,106 patients | 400 patients; 34 events |
| Sensitivity | AUC precision, observed AUC 0.683 | 1,384 patients | 400 patients; 34 events |

The study used all eligible retrospective cases. These calculations are retrospective adequacy/precision assessments and are not presented as prospective sample-size planning.
