# Supplementary material for: Development and validation of a nomogram for predicting chest drainage after CT-guided percutaneous lung biopsy: a multicenter retrospective study
Source: Front Oncol. 2026 Oct 12;16:1930072. doi: 10.3389/fonc.2026.1930072 (PMC13696289; doi:10.3389/fonc.2026.1930072)
Supplement: Supplementary file 3 [file Table3.docx]

## Supplementary Table S3. Five-case online-calculator verification

| **Case** | **Nine model inputs** | **Frozen R probability** | **Web probability** | **Absolute difference** | **Displayed risk** | **Passed** |
| --- | --- | --- | --- | --- | --- | --- |
| Case 1 | Age 20 y; diameter 2 mm; Female; Supine; emphysema No; Upper; LUL; fissure No; pleural adjacency No | 0.018673610997336977 | 0.018673610997336977 | 0 | 1.9% | TRUE |
| Case 2 | Age 60 y; diameter 24 mm; Female; Supine; emphysema No; Upper; LUL; fissure No; pleural adjacency No | 0.037988615535989 | 0.037988615535989 | 0 | 3.8% | TRUE |
| Case 3 | Age 65 y; diameter 30 mm; Male; Prone; emphysema Yes; Upper-middle; LLL; fissure No; pleural adjacency Yes | 0.03958258091541859 | 0.03958258091541859 | 0 | 4% | TRUE |
| Case 4 | Age 70 y; diameter 10 mm; Female; Lateral; emphysema No; Middle; RML; fissure Yes; pleural adjacency No | 0.09087447495066472 | 0.09087447495066477 | 4.163336342344337e-17 | 9.1% | TRUE |
| Case 5 | Age 80 y; diameter 50 mm; Male; Lateral; emphysema Yes; Lower; RLL; fissure Yes; pleural adjacency No | 0.3899291670744326 | 0.3899291670744327 | 1.1102230246251565e-16 | 39% | TRUE |

All cases passed the prespecified absolute numerical tolerance of 10⁻¹⁰. Displayed percentages are rounded to one decimal place.
