# Supplementary material for: Development and validation of a nomogram for predicting chest drainage after CT-guided percutaneous lung biopsy: a multicenter retrospective study
Source: Front Oncol. 2026 Oct 12;16:1930072. doi: 10.3389/fonc.2026.1930072 (PMC13696289; doi:10.3389/fonc.2026.1930072)
Supplement: Supplementary file 4 [file Table4.docx]

## Supplementary Table S4. Frozen ridge-logistic coefficients, coding, and reference categories

| **Predictor** | **Coding/comparison** | **Reference category/unit** | **β coefficient** | **exp(β)** |
| --- | --- | --- | --- | --- |
| Intercept | Baseline log odds | Female; supine; no emphysema; upper location; LUL; no fissure traversal; not pleura-adjacent | -4.3142 |  |
| Age | Per 1-year increase | Continuous, years | 0.0175 | 1.0176 |
| Lesion short-axis diameter | Per 1-mm increase | Continuous, mm | 0.0014 | 1.0014 |
| Sex | Male vs female | Female | 0.4289 | 1.5356 |
| Planned patient position | Prone vs supine | Supine | 0.1333 | 1.1426 |
| Planned patient position | Lateral vs supine | Supine | 0.1896 | 1.2088 |
| Emphysema | Yes vs no | No | 0.5979 | 1.8184 |
| Longitudinal lesion position | Upper-middle vs upper | Upper | 0.1418 | 1.1523 |
| Longitudinal lesion position | Middle vs upper | Upper | -0.1853 | 0.8309 |
| Longitudinal lesion position | Middle-lower vs upper | Upper | 0.1780 | 1.1948 |
| Longitudinal lesion position | Lower vs upper | Upper | 0.2908 | 1.3376 |
| Anatomical lung lobe | LLL vs LUL | LUL | -0.4372 | 0.6459 |
| Anatomical lung lobe | RUL vs LUL | LUL | 0.0170 | 1.0171 |
| Anatomical lung lobe | RML vs LUL | LUL | -0.1264 | 0.8813 |
| Anatomical lung lobe | RLL vs LUL | LUL | -0.0048 | 0.9952 |
| Planned fissure traversal | Yes vs no | No | 0.8956 | 2.4488 |
| Lesion adjacent to visceral pleura | Yes vs no | No | -0.9179 | 0.3994 |

The model uses lambda.1se=0.0325694922143983. The predicted probability is $p=\frac{1}{1+\exp(-LP)}$. exp(β) values are coefficient transformations and are not conventional unpenalized effect estimates.
