# Supplementary material for: Development and validation of a nomogram for predicting chest drainage after CT-guided percutaneous lung biopsy: a multicenter retrospective study
Source: Front Oncol. 2026 Oct 12;16:1930072. doi: 10.3389/fonc.2026.1930072 (PMC13696289; doi:10.3389/fonc.2026.1930072)
Supplement: Supplementary file 5 [file Table5.docx]

## Supplementary Table S5. Penalty selection and sensitivity analysis

| **Analysis** | **Value/reporting role** | **Interpretation** |
| --- | --- | --- |
| Primary penalty rule | lambda.1se = 0.03256949 | Prespecified primary model; stronger shrinkage |
| Alternative penalty rule | lambda.min | Sensitivity analysis only; not selected using external results |
| Cross-validation | Stratified 10-fold | Repeated within every development bootstrap resample |
| Bootstrap repetitions | 1,000 | Complete modeling process repeated |
