# Supplementary material for: Development and validation of a nomogram for predicting chest drainage after CT-guided percutaneous lung biopsy: a multicenter retrospective study
Source: Front Oncol. 2026 Oct 12;16:1930072. doi: 10.3389/fonc.2026.1930072 (PMC13696289; doi:10.3389/fonc.2026.1930072)
Supplement: Supplementary file 6 [file Table6.docx]

## Supplementary Table S6. Actual precision in the external-validation cohort

| **metric** | **estimate** | **ci lower** | **ci upper** | **ci width** |
| --- | --- | --- | --- | --- |
| Outcome proportion | 0.085 | 0.0615 | 0.1164 | 0.054900000000000004 |
| AUC | 0.683 | 0.579 | 0.779 | 0.20000000000000007 |
| Brier score | 0.0755 | 0.055 | 0.0982 | 0.043199999999999995 |
| Calibration intercept | 0.41 | 0 | 0.72 | 0.72 |
| Calibration slope | 1.13 | 0.52 | 1.85 | 1.33 |
| O/E ratio | 1.44 | 1 | 1.89 | 0.8899999999999999 |
