# Supplementary material for: Development and validation of a nomogram for predicting chest drainage after CT-guided percutaneous lung biopsy: a multicenter retrospective study
Source: Front Oncol. 2026 Oct 12;16:1930072. doi: 10.3389/fonc.2026.1930072 (PMC13696289; doi:10.3389/fonc.2026.1930072)
Supplement: Supplementary file 7 [file Table7.docx]

**TRIPOD 2015 Checklist**

| **Item** | **Section/topic** | **How the item is addressed** | **Reported location** |
| --- | --- | --- | --- |
| 1 | Title | Title identifies development and external validation, target population, and chest-drainage outcome. | Title |
| 2 | Abstract | Structured summary includes objective, design, settings, participants, predictors, outcome, analysis, results, and conclusions. | Abstract |
| 3a | Background and objectives | Clinical context and rationale, including prior evidence, are described. | Introduction |
| 3b | Background and objectives | Development and external-validation objectives are explicit. | Introduction |
| 4a | Source of data | Retrospective cohorts and their separate development/validation roles are stated. | Methods—Study design and participants |
| 4b | Source of data | Accrual dates are given for both institutions. | Methods—Study design and participants |
| 5a | Participants | The two participating cancer hospitals and their locations are identified; their development and external-validation roles are described. | Title page; Methods—Study design and participants |
| 5b | Participants | Eligibility and exclusion criteria are listed. | Methods—Study design and participants |
| 5c | Participants | Biopsy and post-procedural monitoring are described. | Methods—Biopsy procedure and outcome |
| 6a | Outcome | Closed chest drainage, assessment timing, threshold, catheter type, and aspiration classification are defined. | Methods—Biopsy procedure and outcome |
| 6b | Outcome | Outcome adjudicators were unaware of model candidate predictors. | Methods—Biopsy procedure and outcome |
| 7a | Predictors | All nine predictors, measurement methods, coding, and prediction time are defined. | Methods—Prediction time and predictors |
| 7b | Predictors | Blinding and repeat-reading procedures for emphysema are reported. | Methods—Prediction time and predictors; Supplementary Table S1 |
| 8 | Sample size | The available-cohort basis and formal Riley assessments for development and external validation are reported. | Methods—Statistical analysis; Results—Model specification; Supplementary Table S2 |
| 9 | Missing data | No missing predictor/outcome data among included patients; complete-record analysis and pre-inclusion exclusions explained. | Methods—Study design and participants ; Results—Participants |
| 10a | Statistical analysis | Linear terms, spline comparison, and dummy coding are described. | Methods—Statistical analysis; Results—Model specification |
| 10b | Statistical analysis | Ridge fitting, lambda selection, full-process bootstrap internal validation, and model freezing are described. | Methods—Statistical analysis and model development; Supplementary Figure S2; Supplementary Table S5 |
| 10c | Statistical analysis | Frozen-model application without refitting/recalibration is described. | Methods—Statistical analysis and model development |
| 10d | Statistical analysis | AUC, Brier score, calibration measures/plots, DCA, and risk distributions are specified. | Methods—Statistical analysis; Results; Table 3; Figures 3-5; Supplementary Figure S3 |
| 10e | Statistical analysis | Institution 2 is explicitly compared with Institution 1 using characteristics and SMDs. | Methods—Statistical analysis; Results—Participants,; Table 1 |
| 11 | Risk groups | No clinical risk categories were created; predicted-risk groups were used only for calibration plots. | Methods—Statistical analysis |
| 12 | Development vs validation | Different institutions, calendar coverage, equipment, reconstruction, lesion size, and planned position are reported. | Methods; Results—Participants; Table 1 |
| 13a | Participants | Flow and outcome counts are reported. | Results—Participants; Figure 1 |
| 13b | Participants | Characteristics and absence of missingness are reported by institution. | Results—Participants; Table 1 |
| 13c | Participants | Not applicable: no univariable screening or data-driven exclusion from the multivariable model. | Not applicable: all nine predictors were prespecified and retained; no univariable screening was used. |
| 14a | Model development | Participants, events, and 16 predictor parameters are stated. | Results—Model specification |
| 14b | Model development | Not performed because predictor retention was prespecified and not based on unadjusted associations. | Not applicable: predictor retention was prespecified and not based on unadjusted associations. |
| 15a | Model specification | The complete intercept, coefficients, coding, reference categories, linear predictor, and probability equation are provided. | Results—Model specification; Table 2; Supplementary Table S4 |
| 15b | Model specification | The nomogram, predictor coding, web calculator, required-input behavior, version information, and five-case verification are provided. | Figure 2; Supplementary Figure S1; Supplementary Table S3; app.R |
| 16 | Model performance | Development and external performance are reported, with external confidence intervals. | Results—Model performance; Table 3; Figures 3-4; Supplementary Table S6 |
| 17 | Model updating | No updating was performed; possible future local recalibration is discussed. | Methods; Discussion |
| 18 | Limitations | Selection, sample size, temporal data, reproducibility, omitted factors, and external scope are discussed. | Discussion—limitations |
| 19a | Interpretation—validation | External performance is interpreted against development case mix and uncertainty. | Discussion |
| 19b | Interpretation—development | Predictive contributions are interpreted without causal claims and in context of prior studies. | Discussion |
| 20 | Implications | Intended use, non-use, and prospective research needs are specified. | Discussion; Conclusion |
| 21 | Supplementary information | Corrected supplementary materials provide emphysema reproducibility, sample-size assessment, calculator verification, frozen coefficients, penalty sensitivity, external precision, and Figures S1-S3; versioned app.R is supplied separately. | Corrected Supplementary Material (Tables S1-S6; Figures S1-S3); app.R |
| 22 | Funding | Funding source is stated. | Funding |
